# Surgical Management of Mandibular Central Incisors with Dumbbell Shaped Periapical Lesion: A Case Report

**DOI:** 10.1155/2014/769381

**Published:** 2014-07-08

**Authors:** Roopadevi Garlapati, Bhuvan Shome Venigalla, Jayaprakash D. Patil, Thumu Jayaprakash, C. H. Krishna Chaitanya, Rama S. Kalluru

**Affiliations:** ^1^Department of Conservative Dentistry and Endodontics, Kamineni Institute of Dental Sciences, Narketpally, Nalgonda, Andhra Pradesh 508254, India; ^2^Department of Conservative Dentistry and Endodontics, Sri Sai College of Dental Surgery, Vikarabad, Andhra Pradesh 501101, India; ^3^Department of Conservative Dentistry and Endodontics, Saint Joseph Dental College, Eluru, Andhra Pradesh 534003, India; ^4^Department of Conservative Dentistry and Endodontics, Sibar Institute of Dental Sciences, Guntur, Andhra Pradesh 522509, India

## Abstract

Dental traumatic injuries may affect the teeth and alveolar bone directly or indirectly. Pulpal necrosis and chronic and apical periodontitis with cystic changes are the most common sequelae of the dental traumatic injuries, if the teeth are not treated immediately. This case report focuses on the conventional and surgical management of mandibular central incisors. A twenty-four-year-old male patient presented with pain in the mandibular central incisors. Radiographic examination revealed mandibular central incisors with dumbbell shaped periapical lesion. After root canal treatment, parendodontic surgery was performed for mandibular central incisors. After one-year recall examination, the teeth were asymptomatic and periapical lesion had healed.

## 1. Introduction

Dental traumatic injuries usually occur in 7- to 12-year-old age group and mostly due to falls and accidents near home or school. Anterior region of the mouth is most commonly affected in dental trauma [1]. Dental trauma may affect the teeth and alveolar bone and may involve the pulp and periodontal ligament directly or indirectly [2].

Dental trauma is one of the factors which is associated with disruption of blood supply to the pulp which is responsible for the occurrence of pulpal necrosis later developing into endodontic infection. Pulpal infection may be immediate or delayed. In young patients with delayed treatment, the pulp may show various responses like internal resorption, dystrophic calcification, and pulpitis which may evolve into partial or total pulpal necrosis [3]. Depending on bacterial and host related factors, endodontic infection progresses and perpetuates into acute or chronic apical periodontitis [4]. Especially, the dental traumatic injuries affecting the anterior teeth can result in pain, psychological problems, and disfigurement of the face and the untreated traumatic teeth may develop cyst like apical periodontitis [5].

To manage such cases, periapical surgery of the affected teeth is one of the treatment options. The success rate achieved by traditional means of surgery varies from 40% to 90%. With the advanced endodontic surgical armamentarium, the success rate increased to 96.8% [6]. Zuolo et al. reported that the postsurgical outcome is 97% for the anterior teeth and 85% for the posterior teeth due to complex radicular anatomy [7].

This case report presents the successful treatment of a “trauma induced large periapical lesion in mandibular central incisors by combined nonsurgical and surgical endodontic treatment” and the case was periodically examined.

## 2. Case Report

**Figure 1:**
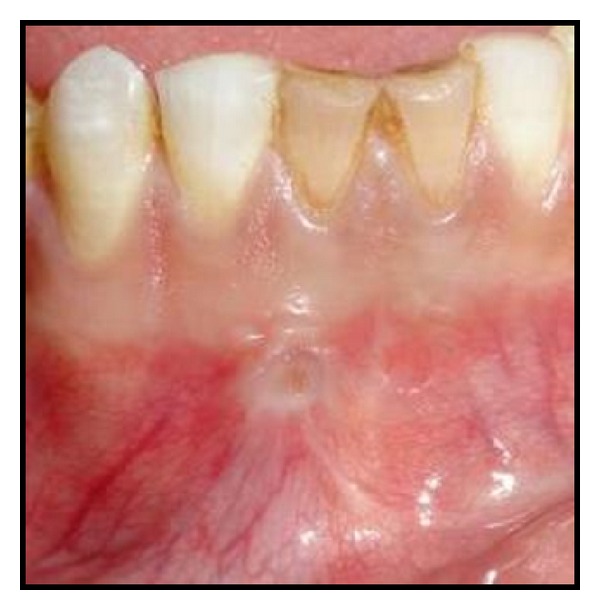
Sinus opening on the labial aspect of Mandibular right central incisors.

**Figure 2:**
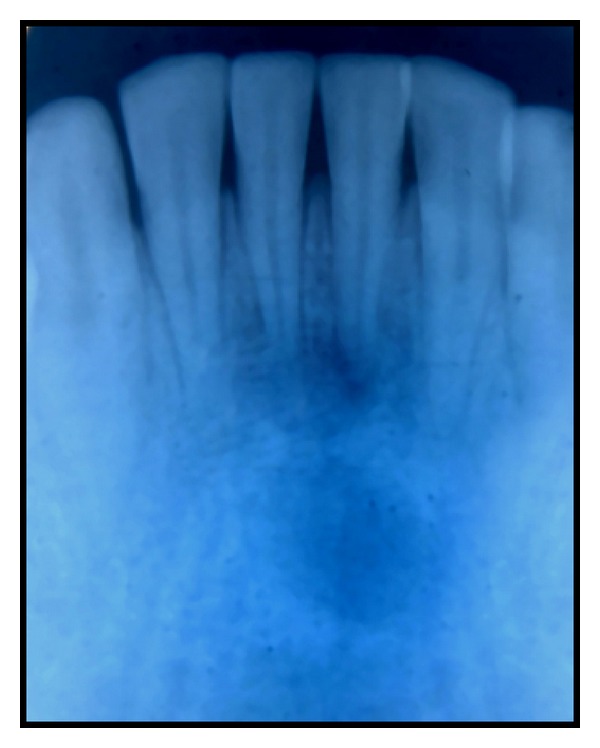
Pretreatment radiograph showing large periapical lesion involving Mandibular central incisors.

**Figure 3:**
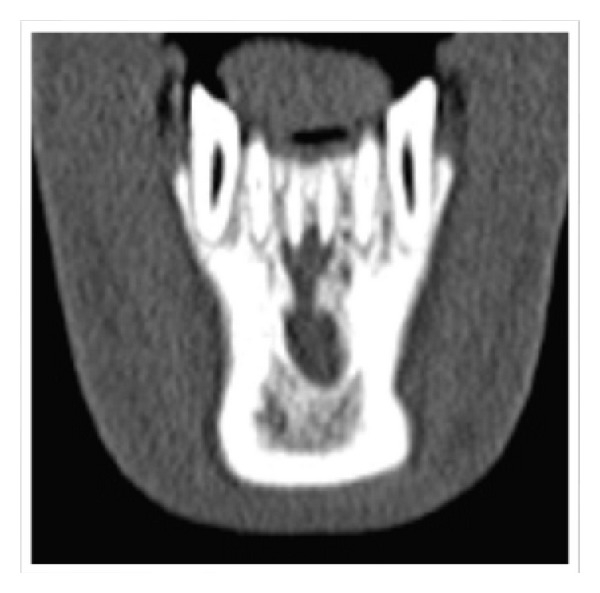
CT Image showing Dumbbell shape periapical lesion in relation to Mandibular central incisors.

A 20-year-old male patient was referred to the Department of Conservative Dentistry and Endodontics with pain and labial swelling in the lower anterior region for two weeks. The pain was continuous and throbbing. His medical history was noncontributory. His dental history revealed trauma to lower anterior teeth due to accident five years ago. On clinical examination, both mandibular central incisors were discolored. Soft tissue examination revealed labial swelling over these teeth; a sinus opening was seen on the labial aspect of mandibular right central incisor. The area was tender on palpation and the teeth were tender on percussion (Figure 1). On vitality examination, teeth were nonvital. Radiographic examination revealed a large dumbbell shaped periapical radiolucency associated with 31, 41 (Figure 2). As dumbbell shaped periapical lesion is a rare occurrence, a computed tomography (CT) scan was advised for 31, 41 to know the exact extension and for the accurate diagnosis of the periapical lesion (Figure 3). The periapical lesion associated with 31, 41, having regular borders, was seen along the apical and lateral root surfaces of mandibular central incisors. The periapical lesion resembles a dumbbell shape and was approximately 2.5 × 1 cm in its greatest dimensions. Based on the above clinical and radiographic findings, mandibular central incisors were diagnosed as having chronic apical periodontitis with cystic changes. A combined approach of orthograde endodontic treatment for 31, 41 followed by periapical surgery was planned. The patient was informed about the procedure and consent was taken.

Local anaesthesia was administered, under rubber dam isolation, and access cavity was done using a number 2 high speed round diamond bur. Working length was determined by apex locator and canal patency was checked with number 15 K-file. Cleaning and shaping of the root canals were performed by hand instruments with step-back technique up to 45 ISO size K-file with alternate irrigation of 3% sodium hypochlorite (NaOCl) solution and saline. Two percent chlorhexidine (CHX) was used as a final irrigating solution. Calcium hydroxide [Ca(OH)_2_] paste (RC cal) was given as an intracanal medicament for one week. Patient was prescribed antibiotics and analgesics to manage pain and swelling. Patient was prescribed 500 mg of amoxicillin thrice a day for five days and combination of 100 mg of aceclofenac and 15 mg of serratiopeptidase twice a day for five days. One week later, the patient was asymptomatic, obturation was done with cold lateral compaction technique, and the access cavity was restored with composite resin. Before performing the endodontic surgery, the patient was advised to undergo blood investigations to rule out bleeding disorders. Complete blood picture and coagulation studies report were normal. The general health condition of the patient before the surgery was good and he fell under ASA I, according to “ASA” physical status classification system.

### 2.1. Surgical Management

**Figure 4:**
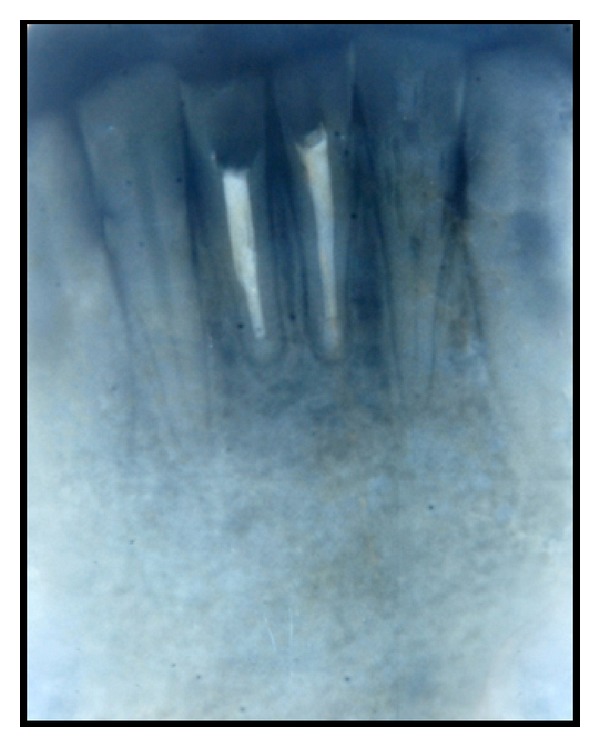
Postoperative radiograph after one year showing healing.

Under local anaesthesia, a full thickness mucoperiosteal flap was elevated. A large soft lesion was seen involving the root apices of 31, 41. The lesion was circumferentially separated from the bony crypt and the teeth. Using gracey curettes, the granulation tissue in the apical and lateral root surfaces of the mandibular central incisors was curetted. For the histopathological examination, the granulation tissue was fixed in 10% buffered formalin. The surgical site was washed with sterile saline solution after the complete removal of the lesion. Apical 3 mm of the roots was resected for 31, 41 and the retrograde filling was done with mineral trioxide aggregate (MTA). As the extension of the defect was large, bone graft (Perioglas) was placed. The mucoperiosteal flap was sutured in place and the periapical radiograph was taken for the confirmation of accuracy of retrograde filling for 31, 41. The granulation tissue was sent for histopathological examination, the findings were suggestive of cystic capsule. The patient was periodically reviewed after 3 months, 6 months, and one year. Patient was asymptomatic during one-year follow-up. At one-year follow-up, a radiograph was taken in relation to mandibular central incisors, which confirmed the satisfactory healing of periapical lesion (Figure 4).

## 3. Discussion

The endodontic management of traumatized mandibular central incisors of a 20-year-old patient was described in this case report. After dental injury, if immediate and appropriate treatment is provided, then it results in successful endodontic outcome. Pulpal response to dental trauma is variable. In some cases, the pulp remains normal, whereas in some cases it becomes necrotic. As a consequence to dental trauma, the pulp loses its ability to protect itself from bacterial invasion and the bacteria penetrates through the dentinal tubules, colonizes in the necrotic pulp, and leads to the development of periapical lesion [2].

Persistent chronic infection can lead to formation of a periapical cyst. Periapical cysts commonly occur in the mandible and may appear as unilocular or multilocular radiolucencies on radiographs. Cystic lesions of the mandible can result in bone remodeling which weakens the bone, leading to functional changes and predisposing the patient to infection and pathologic fracture [8]. Natkin et al. reported that if the radiographic lesion size is 200 mm^2^ or larger, then the incidence of cysts was almost 100% and they have analyzed the data of different studies relating the radiographic lesion size to histology [9].

The possible dumbbell shaped radiographic appearance of the present lesion might be due to the chronicity of the lesion and its extension into the lingual aspect through a possible least resistant path [10]. The dumbbell shaped lesion was mimicking the other radiological entities such as normal anatomical landmarks like Lingual foramen and Lingual fossa, reactive lesions like periapical cemental dysplasia, neurovascular diseases like neurofibroma, neurolemmoma, and haemangiomas, and rarely some metastatic diseases like metastatic carcinomas, eosinophilic granulomas, and Hand-Schuller Christian disease. So, we have advised CT scan for accurate diagnosis of the lesion [11].

The differentiation of periapical cysts and granulomas is very difficult by the traditional radiographs. By the radiographs, only the mesiodistal extent of the pathology can be determined but not the buccolingual extent. With the advent of advanced imaging modalities like computed tomography (CT), cone-beam computed tomography (CBCT), ultrasound with power Doppler flowmetry, and magnetic resonance imaging (MRI), differences in density may permit more accurate preoperative diagnosis [12]. Final diagnosis can be performed by histopathological examination of the tissues taken from the biopsy, which is not possible in nonsurgical treatment cases.

When compared with conventional radiography, CT scan allows visualization of teeth and their surrounding structures in three dimensions. CT is a valuable tool for the presurgical assessment of the teeth, periodontal structures, and proximity of the teeth to adjacent vital structures. With the CT, the true size, location, and extension of the periapical lesion are known and also they can be detected and localized in relation to critical anatomic structures. Velvart et al. conducted a study on 50 patients to detect the apical lesion and its relation to important neighbouring anatomic structures and concluded that CT provides additional 3D information during treatment of apical surgery of mandibular premolars and molars when compared to conventional 2D radiographs [13].

Trope et al. used CT scans for the differentiation of radicular cyst and granulomas and they concluded that cyst can be differentiated from granuloma by density [14]. In case of granulomas, periapical lesions are composed of solid soft tissue, whereas in true cysts, they have semisolid, liquefied cystic areas [12]. From the CT scans, periapical cyst is differentiated from a granuloma by the difference in density of the content of the cyst cavity and granulomatous tissue. Nair and Nair and Cotton et al. reported the use of CT scans for the diagnosis of periapical lesions. Aggarwal et al. reported that CT scans provide an additional and more accurate diagnosis of periapical lesions equal to those of histopathological diagnosis [15].

The main indications of periradicular surgery includes obstructed canals, failed endodontically treated cases, extruded root filling materials, and lesions after traumatic injuries. Apicoectomy, periradicular curettage, and root resection are performed during periapical surgery, for achieving successful outcome [16, 17]. During nonsurgical endodontic treatment, long-term Ca(OH)_2_ therapy is one of the options for treating large cystic periapical lesions. If this treatment fails, then periapical surgery is considered.

The main objective of endodontic treatment is complete eradication of microorganisms from the root canal system. Ca(OH)_2_ was used as an intracanal medicament for a period of one week. Sjogren et al. reported that Ca(OH)_2_ as an intracanal medicament for a period of one week efficiently eliminates the bacteria in the root canals and also causes healing of periapical lesion [18].

In the present case, MTA was used as a root end filling material during periapical surgery of 31, 41. Parirokh and Torabinejad reported that MTA produced cementum formation in 23% of the specimens after 2–5 weeks of periapical surgery and more than 80% of root-end filled cavities with MTA showed deposition of cementum 10–18 weeks after surgery. MTA produces favourable results in terms of absence of inflammation, formation of hard tissue and cementum [19]. Perioglas was used as a bone graft material. Perioglas is an alloplastic, silicate based synthetic bone augmentation material used for filling periodontal defects with bonding and for integration of soft tissue and bone. It is used in addition to conventional surgery during the treatment of intrabony defects. Perioglas has osteoconductive effect and regulates bone formation [20].

During surgical endodontic therapy, necrotic cells, tissue debris, and bacteria in the periapical lesions are completely removed. However, the endodontic surgery is an invasive procedure; if case selection is proper, then surgical debridement is very effective and quite rapid. Compared to nonsurgical endodontic therapy, healing of periapical wound is much faster after endodontic surgery [21]. Radiographs taken 6 months after treatment suggested satisfactory periapical healing. Radiographic signs, like density change within the lesion and trabecular reformation, confirmed healing of the lesion. Both the mandibular central incisors were asymptomatic and also the soft tissues were healthy.

## 4. Conclusion

This case report illustrates the “successful management of large dumbbell shaped periapical lesion of mandibular central incisors with endodontic treatment followed by periapical surgery.” The results confirmed satisfactory healing of the large periapical lesion which responded favourably to successful parendodontic surgery.

## References

[1] Skaare AB, Jacobsen I (2003). Dental injuries in Norwegians aged 7–18 years. *Dental Traumatology*.

[2] Walton RE, Torabinejad M (2002). Management of traumatized teeth. *Principles and Practice of Endodontics*.

[3] Al-Nazhan S, Andreasen JO, Al-Bawardi S, Al-Rouq S (1995). Evaluation of the effect of delayed management of traumatized permanent teeth. *Journal of Endodontics*.

[4] Bergenholtz G (1974). Micro organisms from necrotic pulp of traumatized teeth. *Odontologisk Revy*.

[5] Grossman LI (1967). Origin of microorganisms in traumatized, pulpless, sound teeth. *Journal of Dental Research*.

[6] Kim S, Kratchman S (2006). Modern endodontic surgery concepts and practice: a review. *Journal of Endodontics*.

[7] Zuolo ML, Ferreira MOF, Gutmann JL (2000). Prognosis in periradicular surgery: a clinical prospective study. *International Endodontic Journal*.

[8] Weber AL, Kaneda T, Scrivani SJ, Aziz S, Som PM, Curtin HD (2003). Jaw: cysts, tumors and nontumorous lesions. *Head and Neck Imaging*.

[9] Natkin E, Oswald RJ, Carnes LI (1984). The relationship of lesion size to diagnosis, incidence, and treatment of periapical cysts and granulomas. *Oral Surgery Oral Medicine and Oral Pathology*.

[10] Shear M, Speight PM (1992). *Cysts of the Oral Regions*.

[11] Wood NK, Goaz PW (1997). *Differential Diagnosis of Oral and Maxillofacial Lesions*.

[12] Simon JHS, Enciso R, Malfaz J, Roges R, Bailey-Perry M, Patel A (2006). Differential diagnosis of large periapical lesions using cone-beam computed tomography measurements and biopsy. *Journal of Endodontics*.

[13] Velvart P, Hecker H, Tillinger G (2001). Detection of the apical lesion and the mandibular canal in conventional radiography and computed tomography. *Oral Surgery, Oral Medicine, Oral Pathology, Oral Radiology, and Endodontics*.

[14] Trope M, Pettigrew J, Petras J, Barnett F, Tronstad L (1989). Differentiation of radicular cyst and granulomas using computerized tomography. *Endodontics & Dental Traumatology*.

[15] Aggarwal V, Logani A, Shah N (2008). The evaluation of computed tomography Scans and ultrasounds in the differential diagnosis of periapical lesions. *Journal of Endodontics*.

[16] Von Arx T (2005). Failed root canals: the case for apicoectomy (periradicular surgery). *Journal of Oral and Maxillofacial Surgery*.

[17] Gerhards F, Wagner W (1996). Sealing ability of five different retrograde filling materials. *Journal of Endodontics*.

[18] Sjogren U, Figdor D, Spangberg L, Sundqvist G (1991). The antimicrobial effect of calcium hydroxide as a short-term intracanal dressing. *International Endodontic Journal*.

[19] Parirokh M, Torabinejad M (2010). Mineral trioxide aggregate: a comprehensive literature review. Part III: clinical applications, drawbacks, and mechanism of action. *Journal of Endodontics*.

[20] Palmieri A, Pezzetti F, Spinelli G (2008). PerioGlas regulates osteoblast RNA interfering. *Journal of Prosthodontics*.

[21] Kvist T, Reit C (1999). Results of endodontic retreatment: a randomized clinical study comparing surgical and nonsurgical procedures. *Journal of Endodontics*.

